# Assessing the therapeutic potential of Tirzepatide in modulating inflammatory responses and mitigating acute pancreatitis

**DOI:** 10.1002/ame2.70083

**Published:** 2025-10-20

**Authors:** Razan Alawaji, Mohamed S. Abdel‐Bakky, Hussein M. Ali, Miad A. Aljuhani, Abdulaziz Arif A. Alshammari, Hashim K. Kamal, Maamoun M. K. Khoja, Kholoud Alsehemi, Mennatallah A. Korani, Eman S. Said

**Affiliations:** ^1^ Department of Pharmacology and Toxicology College of Pharmacy, Qassim University Buraydah Saudi Arabia; ^2^ King Salman Medical City Al Madinah Al Munawwarah Saudi Arabia; ^3^ Faculty of Medicine, Department of Biochemistry Al‐Azhar University Assiut Egypt; ^4^ School of Health and Life Sciences University of Glasgow Glasgow UK; ^5^ Faculty of Medicine Fayoum University Fayoum Egypt; ^6^ Faculty of Medicine, Department of Medical Pharmacology Fayoum University Fayoum Egypt

**Keywords:** acute pancreatitis, disease treatment, inflammation, L‐arginine, p‐Akt, Tirzepatide

## Abstract

**Background:**

Acute pancreatitis (AP) is a severe inflammation of the pancreas, marked by elevated enzyme levels, cellular inflammation, and necrosis. Recent studies emphasize the critical role of inflammation in AP progression. Tirzepatide, a multi‐target agonist of glucagon‐like peptide‐1 (GLP‐1) and glucose‐dependent insulinotropic polypeptide (GIP) receptors, has demonstrated notable anti‐inflammatory and metabolic benefits.

**Methods:**

This study explores the therapeutic potential of Tirzepatide in pancreatitis induced by L‐arginine in rats, focusing on enzymatic markers, cytokine profiles, oxidative stress, and histological outcomes. Over 27 days, rats were distributed into Control, Tirzepatide, L‐Arginine, and L‐Arginine + Tirzepatide groups, with the latter receiving L‐Arginine to induce pancreatitis followed by Tirzepatide administration.

**Results:**

L‐Arginine significantly elevated serum amylase, lipase, and inflammatory mediators (IL‐6, IL‐4, and IL‐10), alongside oxidative stress markers and histopathological deterioration. Conversely, the L‐Arginine + Tirzepatide group exhibited reduced lipase and IL‐6 levels, suppressed reactive oxygen species (ROS) generation, and enhanced anti‐inflammatory cytokines IL‐4 and IL‐10. Histopathological analysis revealed reduced necrosis and tissue damage in the L‐Arginine + Tirzepatide group compared to the L‐Arginine group, indicating Tirzepatide's possible protective effects. Immunofluorescence studies further demonstrated increased p‐Akt expression, supporting the role of Tirzepatide in cellular repair and recovery.

**Conclusion:**

These findings highlight Tirzepatide's ability to mitigate pancreatic damage through antioxidant and anti‐inflammatory mechanisms, underscoring its potential as a pharmacological agent for acute pancreatitis.

## Graphical abstract

Tirzepatide: A peptide‐based GLP‐1/GIP receptor agonist, tested on L‐Arginine‐induced pancreatitis. Lipase/Amylase Levels: Initially elevated due to inflammation but normalized with Tirzepatide. Inflammatory Response: Reduction in pro‐inflammatory cytokines (IL‐6) and enhancement of anti‐inflammatory cytokines (IL‐4, IL‐10). Oxidative Stress: Significant reduction in reactive oxygen species (ROS), indicating antioxidant effects. p‐Akt Pathway Activation: Enhanced cellular repair via the PDK/AKT signaling pathway, promoting cell proliferation and survival. Conclusion: Tirzepatide demonstrates a combination of anti‐inflammatory and antioxidant properties, accompanied by a cellular repair mechanism, making it a promising therapeutic option in pancreatitis.

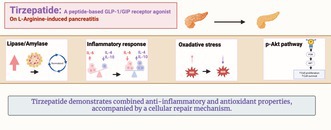

## 1 INTRODUCTION

Acute pancreatitis (AP) is a significant inflammatory‐related condition of the pancreas and one of the most frequent gastrointestinal causes of hospitalization in the United States.^1^ AP results in annual inpatient costs of over $2.6 billion.^2^ It is characterized by tissue edema, inflammatory cell infiltration, and necrosis of acinar cells.^3^ Intracellular activation of digestive enzymes is the primary factor in pancreatitis' pathogenesis. The exocrine component tissue is destroyed in chronic pancreatitis, resulting in endocrine/exocrine insufficiencies, acinar cell atrophy and pancreatic fibrosis. AP is distinguished by inflammatory processes.^3^ In AP, digestive enzymes are elevated while there are low levels of enzymes with severe chronic lesions. These enzymes have been characterized as potent stimulants of increased secretion of pancreatic amylase and lipase.^4^

Cytokines, including tumor necrosis factor‐α (TNF‐α), interleukin‐1 beta (IL‐1β), and interleukin‐6 (IL‐6), are significant inflammatory markers in AP.^5^ IL‐6 is a critical biomarker for evaluating the severity of AP. Compared to other inflammatory indicators, levels of IL‐6 are elevated in the early phase of the disease, suggesting its distinct advantage as an early predictive marker.^6^ IL‐6 may function as an accurate indicator of disease severity, as it provides valuable information about disease progression from the onset while other inflammatory markers rise later.^7^ Conversely, anti‐inflammatory interleukins, including interleukin‐10 (IL‐10), are crucial in the prevention of tissue injury and the limitation of inflammation by suppressing pro‐inflammatory signaling pathways and regulating immune responses.^8^ Interleukin‐4 (IL‐4) is critical for activating a certain type of macrophage. In the presence of IL‐4, macrophages switch to an alternative state in which they stop producing nitric oxide (NO) and deliver antigens to T cells. Instead, they promote tissue healing and the creation of extracellular matrix components. Thus, IL‐4 stimulates an anti‐inflammatory response, which helps to regulate inflammation and facilitates tissue recovery.^9^

It has been reported that reactive oxygen species (ROS) play critical roles in all types of cell death, impacting cellular integrity and function.^10^ ROS are also included in the inflammatory response, where they play a critical role in the initiation and progression of inflammation.^11^ It is well known that pancreatic islet cells demonstrate lower amounts of antioxidant enzymes; thus, the pancreas is more sensitive to oxidative stress than other tissues and organs.^12^ AP is accompanied by an increase in neutrophils, which generate ROS.^5^ Improved antioxidant status has been linked to better clinical outcomes in AP, suggesting that oxidative stress is involved in the early stages of the condition.^11^ Furthermore, AP is typically characterized by stimulating digestive proteases, which are followed by the accumulation of inflammatory cells and the subsequent necrosis of pancreatic tissue.^5^ In rat models, severe necrotizing AP has been induced by the essential amino acid L‐arginine, resulting in a significant influx of inflammatory cells and degenerative alterations in acinar cells.^13^

In addition to mediators of inflammation, oxygen free radicals and NO are critical components in the onset of L‐arginine‐induced pancreatitis, although the precise mechanisms underlying this condition remain unclear.^14^ Various possible mechanisms have been suggested, including changes to cytoskeletons, disruptions to intracellular Ca^2+^ signaling, and malfunctions in the mitochondria and endoplasmic reticulum.^15^ The model currently used to study severe acute necrotizing pancreatitis demonstrates a predictable sequence of events, progressing from pancreatic tissue necrosis to eventual restoration.^3^

Protein kinase B (PKB), commonly known as Akt, is a serine/threonine kinase that plays a critical role in regulating various cellular responses, including glucose and lipid metabolism.^16^ Immune cells are activated by Akt and phosphatidylinositol‐3 kinase (PI3K).^17^ PI3K enhances immune cell activation by promoting the production of the essential anti‐inflammatory cytokine IL‐10 while repressing pro‐inflammatory cytokines. The effects of PI3K/Akt signaling are both significant and far‐reaching.^18^ Three distinct subtypes of Akt have been identified in eukaryotic cells: Akt1 (PKBα), Akt2 (PKBβ), and Akt3 (PKBγ).^19^ Akt1 plays a pivotal role in the Akt signaling pathway, regulating glucose metabolism, apoptosis, cell proliferation, and growth.^20^ Akt2 is predominantly expressed in the liver, brown adipose tissue, and skeletal muscle, where it is critical for the regulation of glucose and lipid metabolic processes in insulin‐responsive cells. In contrast, Akt3 is primarily associated with cellular proliferation, differentiation, apoptosis, and neoplastic growth.^21^

Glucagon‐like peptide (GLP‐1) expression is decreased or completely absent in pancreatic tumor tissues compared to adjacent non‐tumorous pancreatic tissues. The absence of GLP‐1 expression is frequently observed in advanced tumors with lymphatic metastasis and greater diameters and closely correlates with poor prognosis. Studies have demonstrated that GLP‐1 activation has an anti‐cancer effect on pancreatic carcinoma by inhibiting the PI3K/Akt signal transduction pathway. This supports the hypothesis, as mentioned earlier, that GLP‐1‐based therapies may act protectively in the context of pancreatic cancer, especially in individuals with type 2 diabetes, rather than having an adverse effect.^22^ Newly developed as a “twincretin,” Tirzepatide is the first medicine of its type and functions as a dual agonist for the GLP‐1 and glucose‐dependent insulinotropic polypeptide (GIP) receptors simultaneously. It has demonstrated extraordinary effects in considerably lowering blood glucose levels, increasing insulin sensitivity, causing a 20% reduction in body weight, and improving lipid metabolism.^23^

Case reports and animal studies suggest an increased risk of pancreatitis associated with GLP‐1 based therapies.^24^ Nonetheless, GLP‐1 receptor agonists have been associated with increased levels of lipase and amylase. Importantly, these enzyme elevations do not indicate a heightened risk of AP.^25^ Notably, various clinical trials have demonstrated that Tirzepatide, a dual GLP‐1 and GIP receptor agonist, is not associated with an increased risk of pancreatitis in individuals with type 2 diabetes and obesity. These findings provide reassurance regarding the safety profile of Tirzepatide and confirm its appropriateness for long‐term use in managing both glycemic control and weight reduction.^26^

This study was designed to investigate the effect of Tirzepatide on L‐arginine‐induced pancreatitis, aiming to provide deeper insights into its safety profile and potential therapeutic implications.

## 2 MATERIALS AND METHODS

### 2.1 Drugs and antibodies

L‐Arginine monohydrochloride (reagent grade ≥ 98%, HPLC) powder was obtained from Sigma‐Aldrich. Tirzepatide was prepared using the Mounjaro pen supplied by Eli Lilly. Mouse monoclonal p‐Akt and IL‐10 antibodies were sourced from Santa Cruz Biotechnology (TX, USA). Alexafluor 488‐conjugated goat anti‐mouse and Cyanin red (Cy3)‐conjugated goat anti‐mouse secondary antibodies were purchased from Jackson Immuno‐research (PA, USA).

### 2.2 Animals

Forty male rats, weighing between 200 g and 300 g, were obtained from Qassim University's Animal House for this study. The animals were housed in clean, well‐ventilated cages under optimal environmental conditions. They were provided with a balanced diet and free access to water. Ambient temperature was maintained at 20℃–26℃ (68°F–78°F), with humidity levels between 30% and 70%. Lighting followed a 12‐h light and dark cycle. All experiments were conducted under the guidelines of the NIH and Saudi Arabian regulations.^27^

### 2.3 Experimental protocol

The rats were randomly distributed into four groups of ten as follows. Control group (Group 1): This group received distilled water orally throughout the duration of the study. Tirzepatide group (Group 2): This group received subcutaneous (s.c.) injections of 15 mg of Tirzepatide in 50 mL prepared in 0.9% normal saline and adjusted to pH 7. Each rat was administered injections at a dose of 1.55 mg/kg every 3 days over a span of 22 days, totaling eight doses.^28^ L‐Arginine group (Group 3): This group was administered two intraperitoneal (i.p.) injections of L‐arginine at 2.5 g/kg.^29^ L‐Arginine + Tirzepatide group (Group 4): This group was first given two (i.p.) injections of L‐arginine using a dose of 2.5 g/kg, 1 h apart, at the start of the experiment. Once pancreatitis was established, the rats were treated with Tirzepatide injections at the same dosage and schedule described for Group 3.

### 2.4 Preparation of blood and tissue samples

Sampling was conducted at four intervals during the experiment: before starting, on day 4, on day 8, and at the end of the study. The experimental rats received an i.p. injection of 50 mg/kg thiopental sodium for anesthesia. Blood samples were collected from the retro‐orbital venous plexus, placed into tubes, and left for 20 min for coagulation. Extraction of the serum was performed by centrifugation of the sample at 3000 rpm for 15 min and storage at −80°C until assay time. For pancreatic tissue collection, the samples were directly soaked in 10% buffered formalin for 48 h to ensure proper fixation. Following fixation, the specimens underwent a standard histological protocol, including dehydration in alcohol solutions with different grades, clearing with xylene, and embedding in paraffin wax. Thin sections, 4 μm in thickness, were prepared and subjected to Hematoxylin and Eosin (H&E) staining for histopathological examination.

### 2.5 Enzyme‐linked immunosorbent assay (ELISA) for blood analysis

IL‐6, IL‐4, ROS, amylase and lipase were quantitatively analyzed using the ELISA technique following the manufacturer's directions, and sample absorbance was measured at a wavelength of 450 nm. The sample concentration was determined using a standard curve. Serum amylase and lipase were estimated using the double antibody sandwich technique with rat amylase and rat lipase ELISA Kits. Both kits were provided by Mybiosource (CA, USA). Additionally, IL‐6 and IL‐4 levels were measured using sandwich enzyme immunoassays from Cloud‐Clone Corp (TX, USA). Finally, ROS levels were determined using a sandwich enzyme immunoassay from ABclonal (MA, USA).

### 2.6 Pancreas sectioning and staining for histopathological examination

The pancreatic tissues were immersed in 10% formalin solution and fixed in paraffin blocks. Tissues imbedded in paraffin were dehydrated using gradient alcohol concentrations (50%, 70% for 24 h, 90%, and 100% for 12 h) and then defatted in xylene for 2 h. Serial sections of tissues, each 4 μm thick, were cut out for H&E and immunofluorescence techniques.

### 2.7 Hematoxylin and Eosin (H&E) staining and scoring of the pancreatic acinar cell necrosis

Hematoxylin and Eosin (H&E) staining was applied to evaluate morphological and structural changes in the pancreatic tissue samples. This was performed according to Geoffrey's method (2012). In brief, tissues were heated at 58°C for 20 min, deparaffinized using xylene, and rehydrated using different grades of alcohol concentrations. Nuclear staining was performed using Hematoxylin for 5 min, followed by a 15‐s wash. Cytoplasmic staining utilized Eosin for 5 min. Sections were dehydrated using alcohol, cleaned with xylene and finally mounted with mounting media.^30^

Histological scoring of pancreatic acinar cell necrosis in the animal tissues was achieved using H&E stain. The level of necrosis, infiltration of inflammatory cells, oedema and hemorrhage was scored as follows: 0 = absent, 1 = mild, 2 = moderate, 3 = severe, and 4 = very severe.^31^ Five high‐magnification fields were analyzed for each rat section..

### 2.8 Immunofluorescent analysis

**FIGURE 1:**
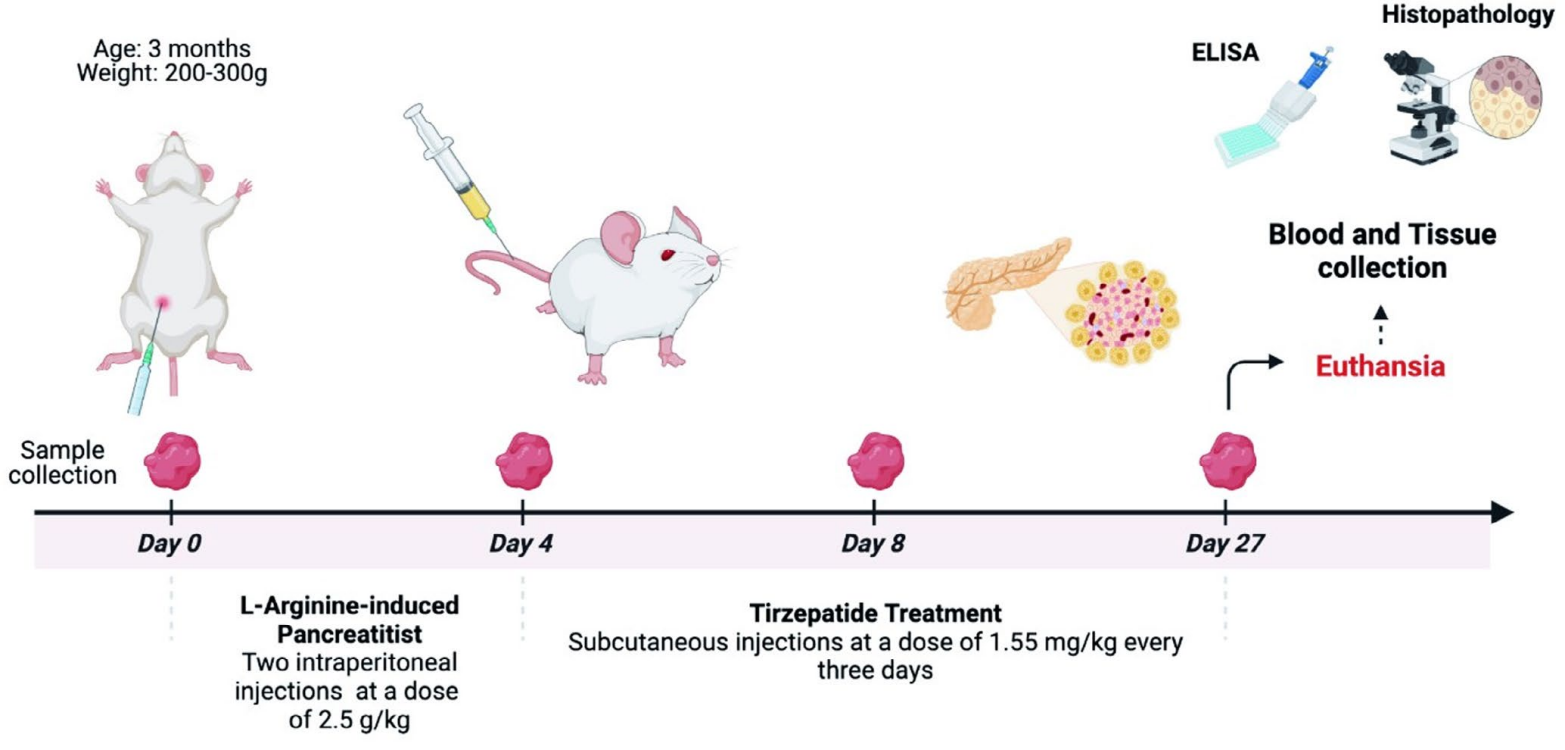
Experimental design timeline for L‐arginine‐induced pancreatitis and Tirzepatide treatment in rats. Male rats were subjected to L‐Arginine‐induced pancreatitis starting on day 0, receiving two intraperitoneal injections of 2.5 g/kg administered 1 h apart. Tirzepatide treatment commenced on day 4, with subcutaneous injections at a dose of 1.55 mg/kg every 3 days until day 27. Blood and tissue samples were collected for ELISA and histopathology analysis, and the animals were euthanized for final assessments.

Both IL‐10 and p‐Akt expression were analyzed using the immunofluorescence technique following the modified procedure of Abdel‐Bakky et al. In brief, tissue sections were processed as previously described. Antigen retrieval was conducted by boiling the sections in citrate buffer (pH 6.0) in a microwave oven at 500 watts for 20 min. The slides were washed with a solution containing 0.05% Tween in phosphate‐buffered saline, and subsequently fixed with methanol in a humidified chamber for 10 min. Tissue sections were subsequently incubated for 1 h. with a blocking solution (1% BSA, 10% horse serum in PBS). The samples were incubated with the primary antibodies (mouse monoclonal IL‐10, and p‐AKT antibodies) for 3 h at 37℃, followed by overnight incubation at 4℃. Subsequent to washing, the slides were incubated with goat anti‐mouse secondary antibodies conjugated with Cyanine red (Cy3) or Alexa 488 at 37℃ for 30 min. Slides were counterstained with 4′,6‐diamidino‐2‐phenylindole (DAPI) to mark the nuclei, washed again, and mounted with coverslips using Fluoromount® mounting solution from DAKO (CA, USA). Images were captured using a Leica fluorescence microscope (Model: Leica DM 5500B, Leica Microsystems, Wetzlar, Germany), obtaining pictures in blue, green and red channels. Quantitative fluorometric analysis was conducted on a minimum of six fields per tissue section utilizing Image‐J software (NIH, USA)^32^ (Figure 1).

### 2.9 Statistical analysis

GraphPad Prism version 10 was used for statistical analysis, with a one‐way ANOVA followed by Tukey's multiple comparison test. Descriptive statistics were performed using mean ± standard deviation (SD). A value of *p* < 0.05 was considered statistically significant.

## 3 RESULTS

### 3.1 Temporal dynamics of lipase and amylase levels across treatment groups

**FIGURE 2:**
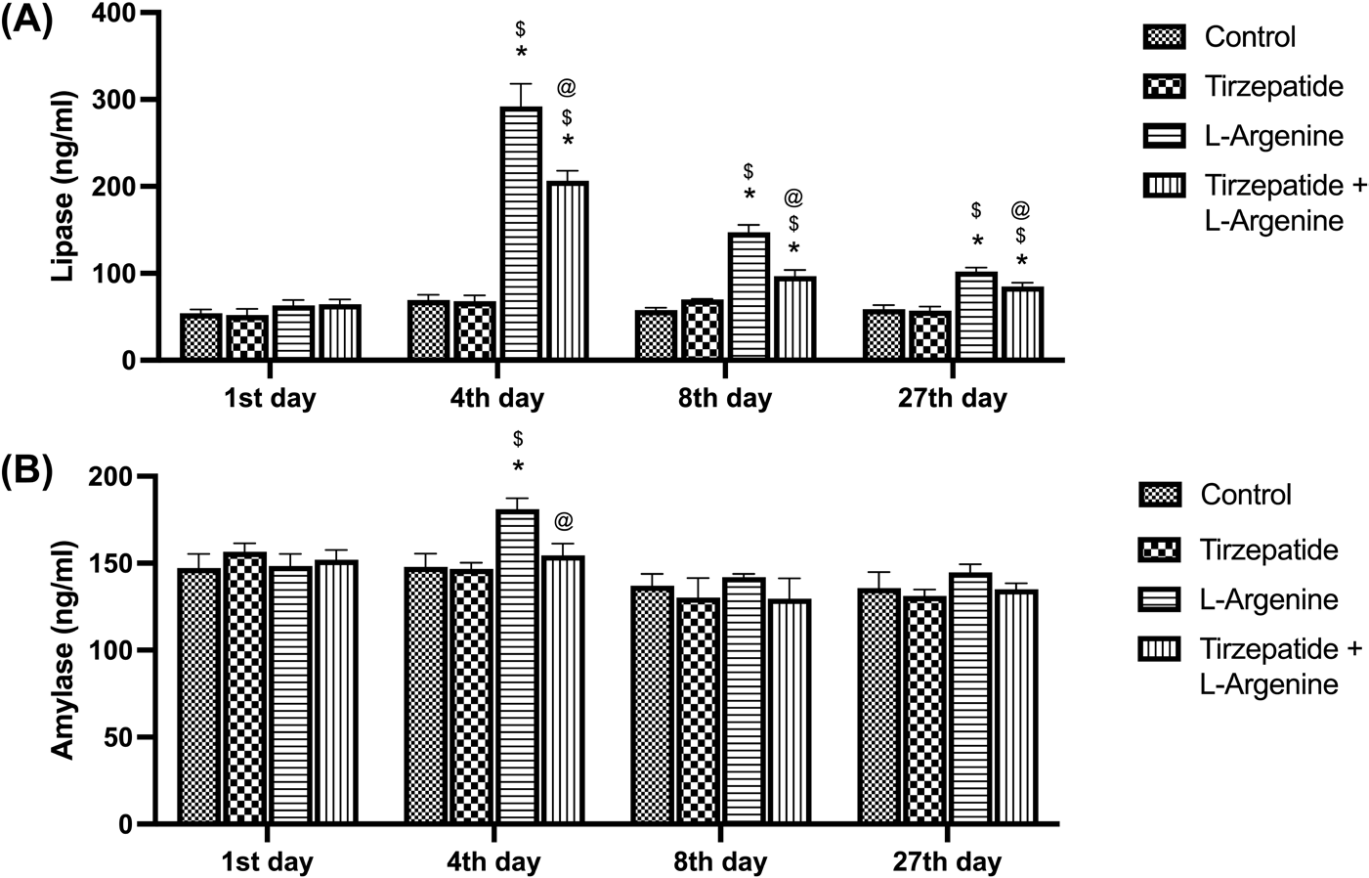
Temporal dynamics of lipase and amylase levels in the different groups. (A) Serum lipase. (B) Serum amylase. Data are expressed as mean ± SD. *p< 0.05 compared to the control group. ^$^ *p* < 0.05 compared to the Tirzepatide group. ^@^ *p* < 0.05 compared to the L‐Arginine group.

This study evaluated temporal changes in lipase and amylase levels across different treatment groups. On day 4, lipase levels in the L‐Arginine group showed a significant increase compared to the Control and Tirzepatide groups. Similarly, the group treated with both L‐Arginine and Tirzepatide exhibited elevated lipase levels relative to the Control and Tirzepatide groups but demonstrated a significant reduction compared to the L‐Arginine group alone (Figure 2A). These trends were consistent on days 8 and 27. Regarding amylase levels, the L‐Arginine group displayed a significant elevation on day 4 compared to the Control and Tirzepatide groups. while the combined treatment group showed a significant reduction relative to the L‐Arginine group. By days 8 and 27, no significant differences in amylase levels were observed among the groups (Figure 2B).

### 3.2 Variations in inflammatory markers (IL‐6, IL‐4, and IL‐10) over time

**FIGURE 3:**
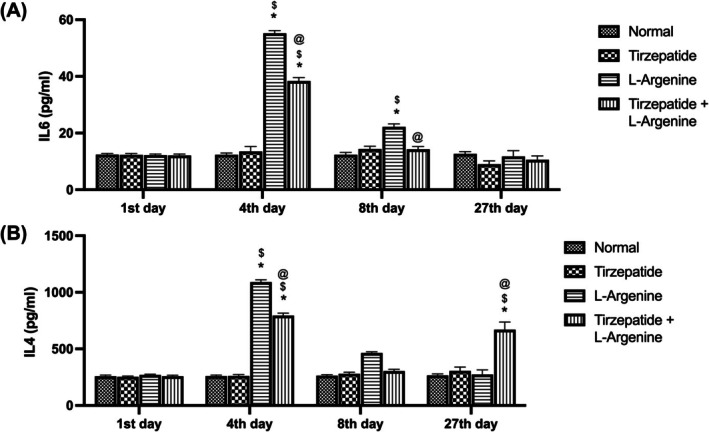
Variations in inflammatory markers over time. (A) Pro‐inflammatory IL6. (B) Anti‐inflammatory IL4. Data are expressed as mean ±SD. *p< 0.05 compared to the control group. ^$^ *p* < 0.05 compared to the Tirzepatide group. ^@^ *p* < 0.05 compared to the L‐Arginine group.

The findings on IL‐6 levels revealed a significant increase in the L‐Arginine and L‐Arginine + Tirzepatide groups compared to the Control and Tirzepatide groups on day 4. However, the L‐Arginine+ Tirzepatide group showed a significant reduction in IL‐6 levels compared to the L‐Arginine group. By day 8, only the L‐Arginine group exhibited a significant elevation in IL‐6 compared to the Control and Tirzepatide groups, while the L‐Arginine + Tirzepatide group displayed a significant decline relative to the L‐Arginine group. On the final day of the experiment (day 27), no significant differences in IL‐6 levels were observed among the groups (Figure 3A).

For IL‐4 levels, significant increases were observed on day 4 in the L‐Arginine and L‐Arginine + Tirzepatide groups compared to the Control and Tirzepatide‐alone groups. The L‐Arginine + Tirzepatide group also demonstrated a significant suppression of IL‐4 levels compared to the L‐Arginine group. On day 27, the L‐Arginine + Tirzepatide group showed a notable increase in IL‐4 levels compared to all other groups, while no significant changes were observed in the other groups. Similarly, on day 8, no significant variations in IL‐4 levels were observed between groups (Figure 3B).

**FIGURE 4:**
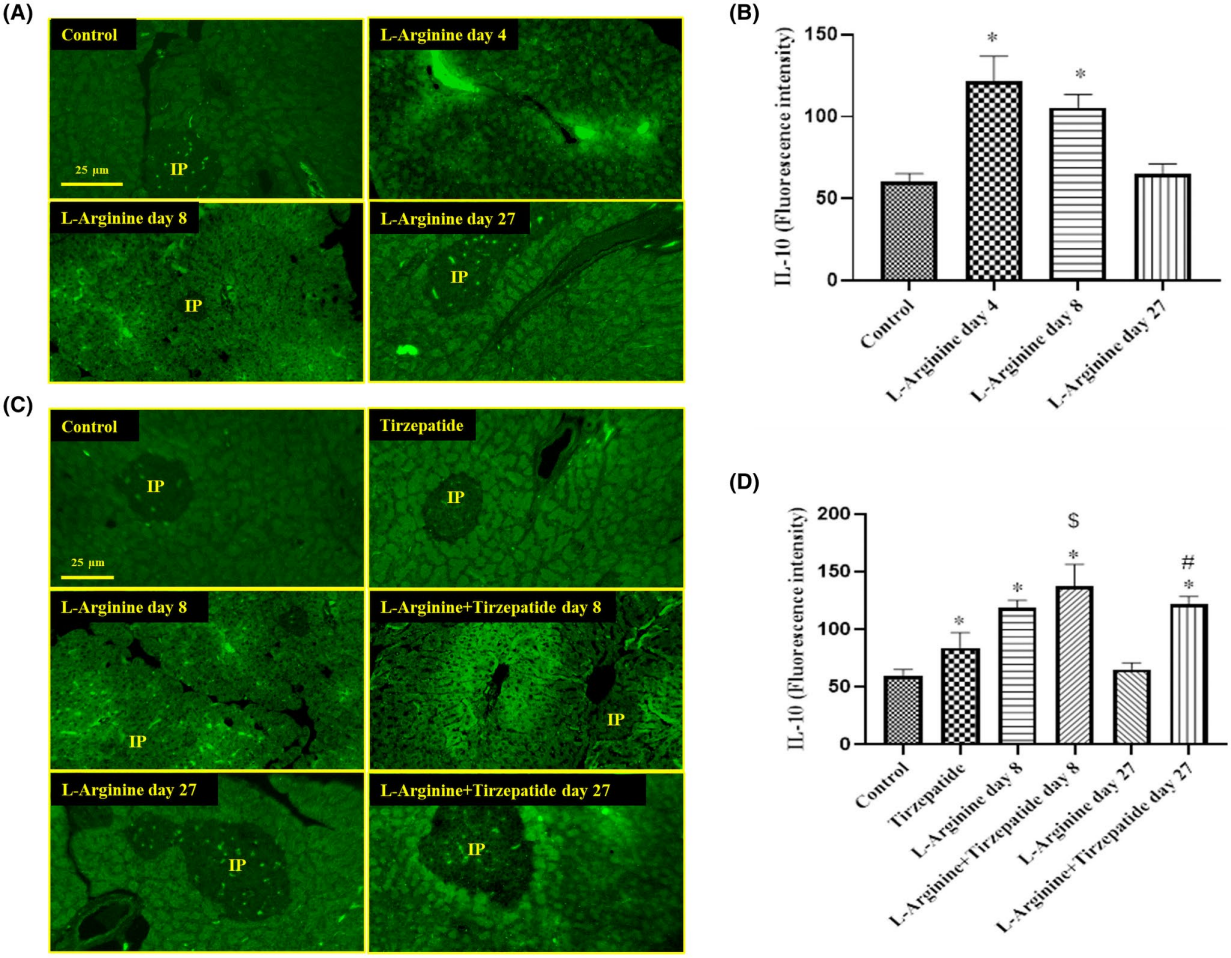
IL‐10 protein expression in the pancreatic tissue sections. (A) Rats were treated with L‐arginine for 4, 8, and 27 days. (B) IL‐10 fluorescence intensities. (C) Rats were treated with L‐arginine in the presence or absence of Tirzepatide. (D) IL‐10 fluorescence intensities. Data are presented as mean ± SD. *p < 0.05 compared to the control group, ^$^ *p* < 0.05 compared to the L‐Arginine group on day 8. ^#^ *p* < 0.05 compared to the L‐Arginine group on day 27 (magnification for both A & C: 400×). IP denotes pancreatic islets.

Regarding IL‐10 expression, measured using immunofluorescence analysis, the treatment with L‐Arginine significantly increased IL‐10 levels on day 4 (in both the pancreatic acinar cells and islets) and day 8 (in the acinar cells) compared to the Control group. By day 27, the L‐Arginine group showed no significant difference in IL‐10 expression compared to the Control group (Figure 4A,B). On the other hand, on day 8 the L‐Arginine + Tirzepatide group displayed a significant increase in IL‐10 expression in both acinar cells and pancreatic islets compared to the L‐Arginine group. On day 27, IL‐10 expression in the Control, Tirzepatide, and L‐Arginine groups remained at basal levels, while the L‐Arginine + Tirzepatide group showed a significant increase in IL‐10 expression in both the acinar cells and islets compared to sections from Control and L‐Arginine at day 27 (Figure 4C,D).

### 3.3 Changes in reactive oxygen species (ROS) across treatment groups

**FIGURE 5:**
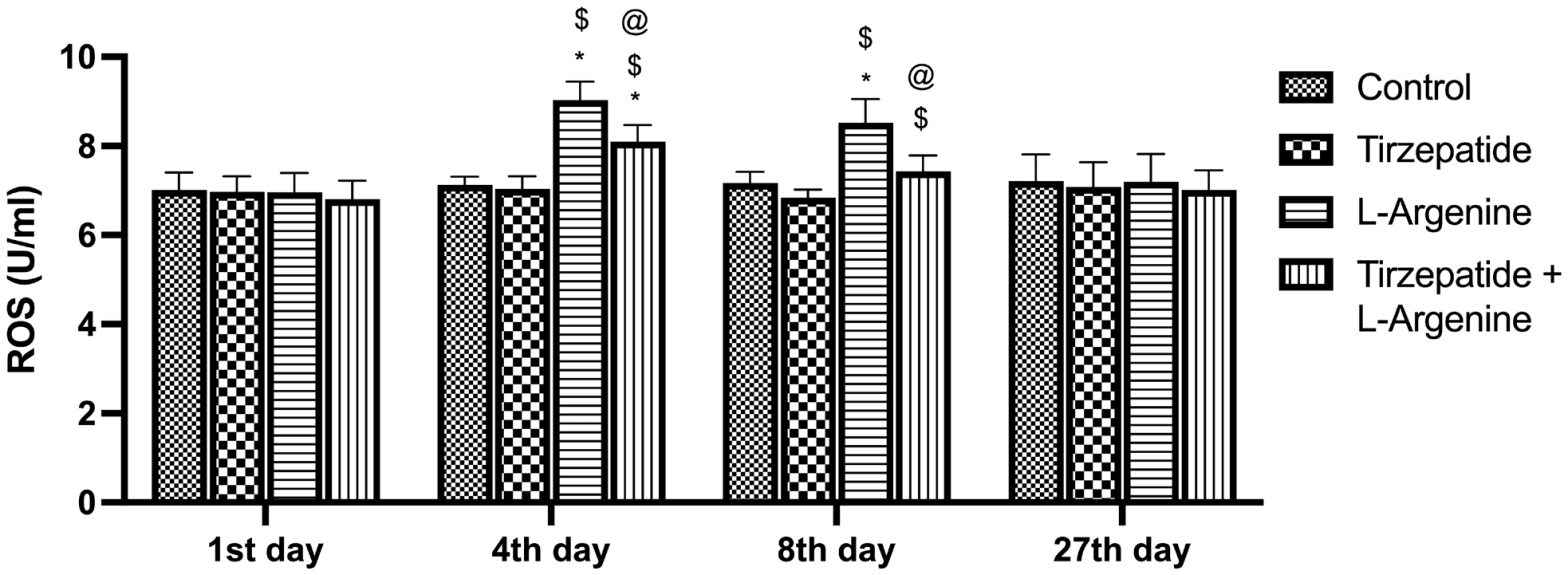
Changes in reactive oxygen species (ROS) across treatment groups. Data are expressed as mean ± SD. *p < 0.05 compared to the control group. ^$^ *p* < 0.05 compared to the Tirzepatide group. ^@^ *p* < 0.05 compared to the L‐Arginine group.

Reactive oxygen species (ROS) levels demonstrated significant variations across the study groups. On day 4, both the L‐Arginine and L‐Arginine + Tirzepatide treatment groups exhibited a significant increase in ROS levels compared to the Control and Tirzepatide groups. However, the L‐Arginine + Tirzepatide treatment group showed a notable reduction in ROS levels compared with the L‐Arginine group, while no significant differences were observed between the Control and Tirzepatide groups. By day 8, the L‐Arginine group displayed a significant elevation in ROS levels relative to the Control and Tirzepatide groups. However, the L‐Arginine + Tirzepatide treatment group showed a significant increase in ROS levels compared to the Tirzepatide group but a decline compared to the L‐Arginine group, with no significant differences observed between the L‐Arginine + Tirzepatide treatment group and the Control group. By day 27, ROS levels showed no significant differences among any of the study groups, indicating a resolution of oxidative stress over time (Figure 5).

### 3.4 Histopathological examination of pancreatic tissue in response to L‐arginine and Tirzepatide treatments

**FIGURE 6:**
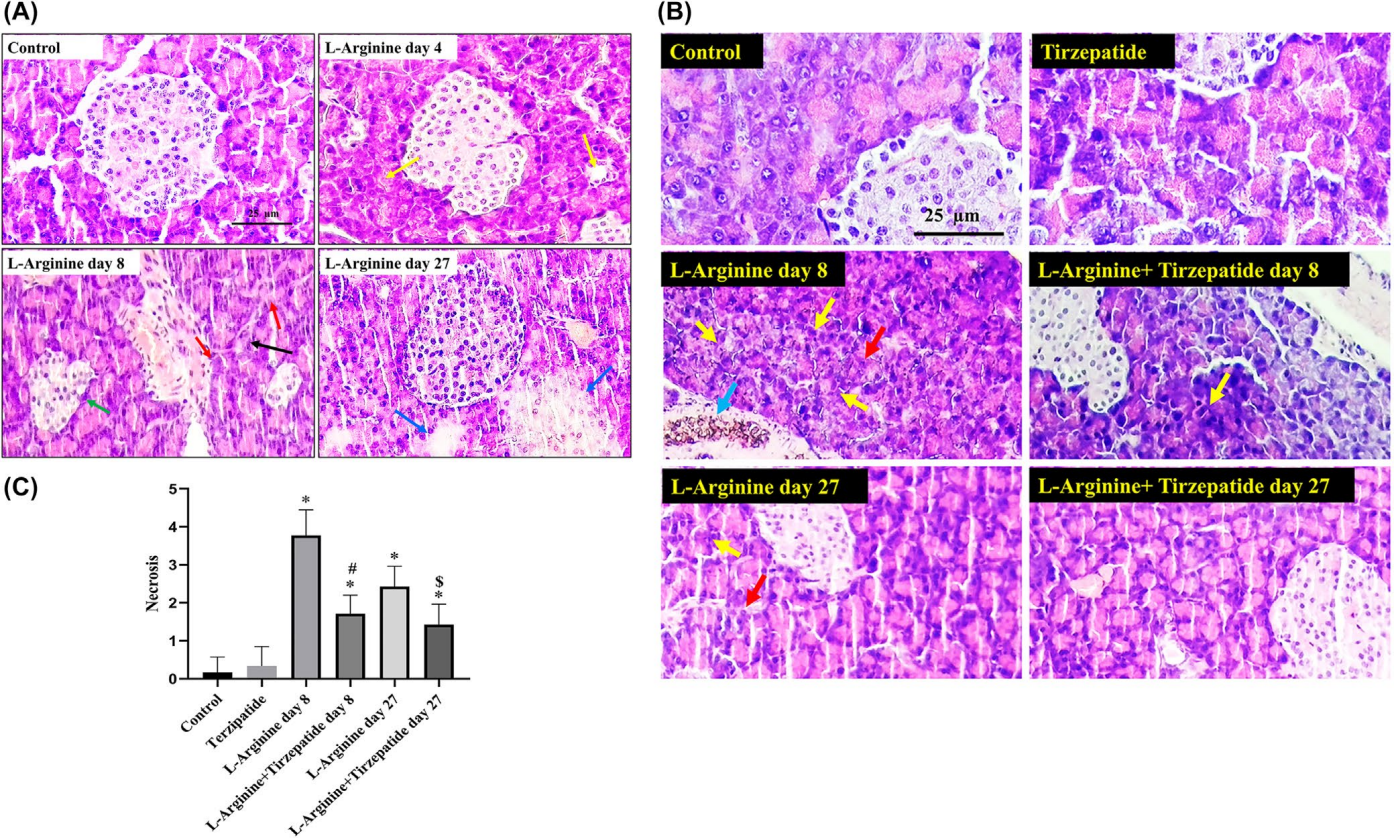
Photomicrographs of histopathological sections stained with Hematoxylin and Eosin (H&E). (A) H&E‐stained pancreatic tissue sections from control and L‐Arginine groups at various time points. (B) H&E‐stained pancreatic tissue sections from control and L‐Arginine groups, with or without Tirzepatide treatment. (C) Histological scores of pancreatic acinar necrosis are shown. Data are presented as mean ± SD. *p < 0.05, significantly different compared to the control group; ^#^ *p* < 0.05, significantly different compared to the L‐Arginine group on day 8; ^$^ *p* < 0.05, significantly different compared to the L‐Arginine group on day 27. Magnification for both A & B: 400×.

The histopathological analysis of pancreatic tissue sections revealed distinct changes across the study groups. Rats of the Control group exhibited well‐preserved pancreatic islets and acinar structures, indicative of normal histology. By contrast, L‐Arginine‐ rats showed evidence of vacuolar degeneration (yellow arrows) on day 4, while those treated for 8 days displayed mild periductal fibrosis (red arrows), and smaller and distorted pancreatic islets (green arrows). By day 27, L‐Arginine rats demonstrated focal areas of lytic necrosis (blue arrows) (Figure 6A).

Pancreatic sections from Control and Tirzepatide rats consistently displayed intact histological features, maintaining normal islet and acinar architecture. However, L‐Arginine rats presented with focal hyperplasia of pancreatic duct epithelium (blue arrows) and pyknotic nuclei (red arrows) on days 4 and 27. Importantly, rats in the L‐Arginine + Tirzepatide group demonstrated notable improvement in histological features, with reduced L‐arginine‐induced damage, suggesting a protective effect of Tirzepatide (Figure 6B,C).

### 3.5 Expression of p‐Akt in pancreatic tissue across treatment groups

**FIGURE 7:**
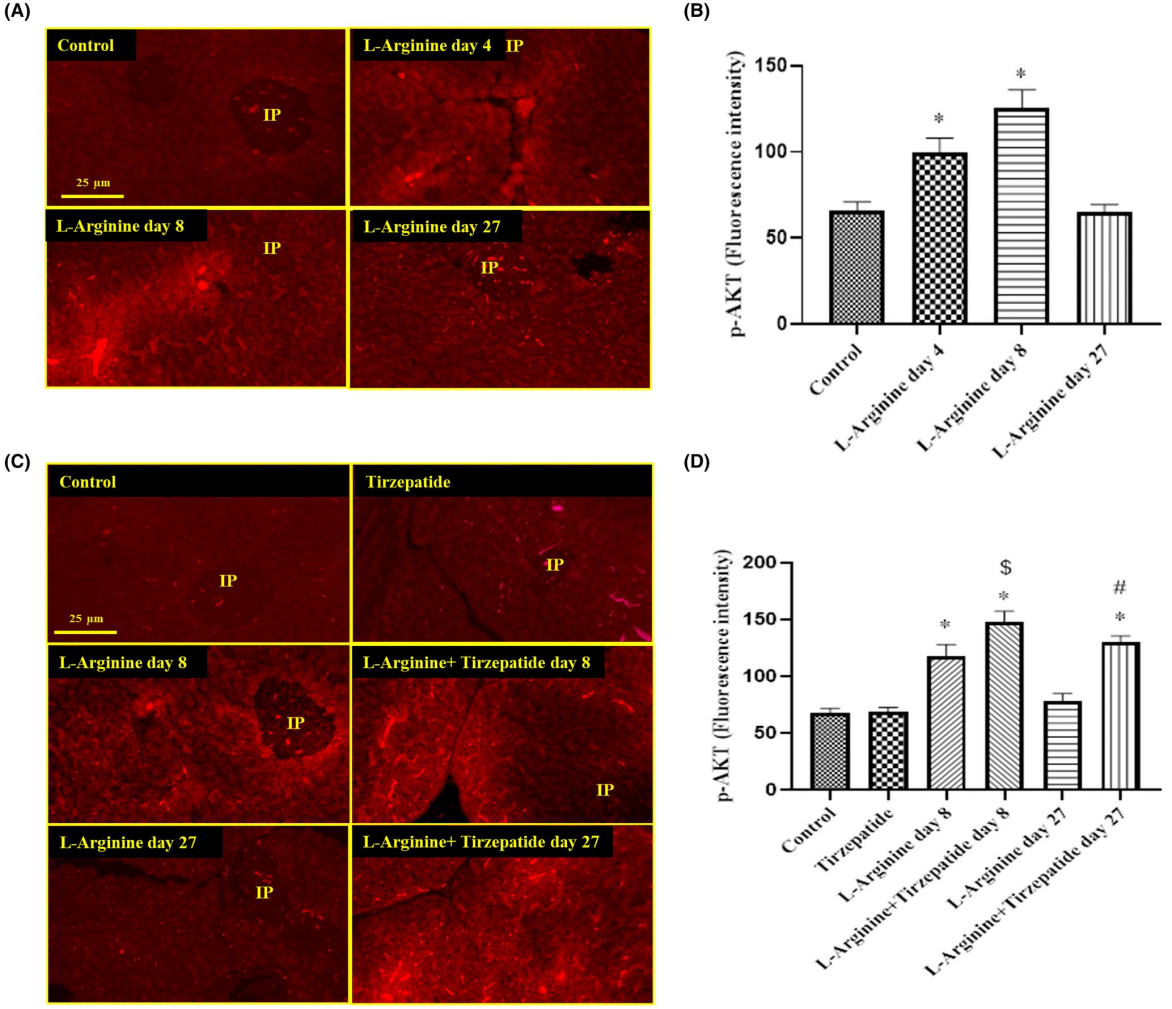
Expression of p‐Akt protein in the tissues of the pancreas. (A) The rats were treated with L‐arginine for 4, 8, and 27 days. (B) p‐Akt fluorescence intensities. (C) The rats were treated with L‐arginine in the presence or absence of Tirzepatide. (D) p‐Akt fluorescence intensities. Data are presented as mean ± SD. *p < 0.05 compared to the control group; ^$^ *p* < 0.05 compared to the L‐Arginine group on day 8; ^#^ *p* < 0.05 compared to the L‐Arginine group on day 27 (magnification for both A & C: 400×). IP denotes pancreatic islets.

The expression of p‐Akt demonstrated distinct patterns across the study groups. In the **L‐Arginine rats**, a significant increase in p‐Akt expression was detected in both the pancreatic acinar cells and islets after 4 and 8 days of treatment compared to the Control group. However, no differences in p‐Akt expression were noted in the L‐Arginine group after 27 days, which showed levels comparable to the Control group (Figure 7A,B).

In the **Control and Tirzepatide groups**, p‐Akt expression remained at basal levels after **27 days**. Co‐treatment of the L‐Arginine + Tirzepatide group for 8 days led to a significant increase in p‐Akt protein expression in both the acinar cells and pancreatic islets compared to the L‐Arginine group at the same time point. Furthermore, co‐treatment of the L‐Arginine + Tirzepatide group for 27 days resulted in a significant elevation of p‐Akt expression in the pancreatic acinar cells compared to the L‐Arginine group at the same time point (Figure 7C,D).

## 4 DISCUSSION

Pancreatitis is characterized by vacuole formation in the cytoplasm, acinar cell necrosis, edema, and infiltration of inflammatory cells.^33^ Despite recent advances in understanding pancreatitis pathophysiology and the development of new treatment solutions, illness management, particularly severe acute pancreatitis (SAP), remains inadequate and is linked with a high death rate.^34^ Furthermore, no drug has been globally approved for the ameliorative treatment or prevention of complications in AP.^35^

This study highlights the differential effects of L‐arginine and Tirzepatide on pancreatic enzyme levels. The marked increase of lipase and amylase levels with L‐arginine administration suggests it may influence metabolic pathways that affect pancreatic function. By contrast, Tirzepatide alone did not significantly increase these enzymes, indicating that it may not have a substantial direct effect on pancreatic enzyme elevation in this context. The significant decrease in enzyme levels following treatment with a combination of Tirzepatide and L‐arginine is particularly interesting, as it suggests a potential interaction between the two compounds that may mitigate any potential pancreatic impact.

It is worth noting that serum lipase often remains elevated slightly longer than amylase due to differences in clearance kinetics. This observation has been described in previous studies, and it helps to explain the discrepancy between the time courses of these enzymes in acute pancreatitis.^36^

In contrast to the findings of this study, prior research has shown that Tirzepatide alone can elevate pancreatic exocrine enzymes, in particular lipase and amylase. However, these elevations were deemed clinically insignificant and did not correspond to a substantial higher risk of acute pancreatitis (AP). This aligns with evidence supporting the pancreatic safety of Tirzepatide, as the observed enzyme changes were minor and did not translate into adverse clinical outcomes.^37^

Notably, a recent large‐scale retrospective cohort study based on data from TriNetX reported that the use of Tirzepatide and semaglutide was associated with a decreased risk of recurrent acute pancreatitis among individuals with type 2 diabetes or obesity and a history of pancreatitis. This finding further supports the possibility that Tirzepatide may exert protective effects against pancreatic inflammation in clinical settings.^38^

According to our study results, L‐arginine significantly elevated IL‐6 levels at earlier time points, while co‐treatment with Tirzepatide moderated these elevations. By the end of the experiment, IL‐6 levels had normalized across all groups. The combination of L‐arginine and Tirzepatide showed early suppression of IL‐4 levels (Day 4) compared to L‐arginine alone, following by late‐stage enhancement (Day 27), highlighting the time‐dependent immunomodulatory effects of the treatment. We also found that Tirzepatide increased IL‐4 and IL‐10 levels during inflammation, which suggests that it is involved in modulating pro‐inflammatory states, indicating a possible clinical application. Moreover, the results of this study demonstrate insights into the dynamic effects of L‐arginine and Tirzepatide on ROS.

The pathogenesis of pancreatitis is influenced by oxidative stress, as excess ROS induces cell death.^39^ These results are consistent with prior research indicating that, by balancing inflammatory and oxidative pathways, Tirzepatide mitigates oxidative stress and exhibits notable anti‐inflammatory properties, potentially offering therapeutic value through these two pathways.^40^, ^41^ Reducing pro‐inflammatory substances like IL‐6 while lowering ROS and inflammatory markers underscores its potential in addressing inflammation and oxidative stress in pathological conditions.^42^

Elevated IL‐6 levels enhance the inflammatory response in AP,^43^ suggesting its crucial role as a biomarker and treatment target for disease severity and outcomes.^44^ Modulating cytokine signaling is crucial for improving therapeutic outcomes, as IL‐4 decreases pro‐inflammatory cytokine production. Targeting IL‐4 pathways could improve inflammation management.^45^ IL‐10, which inhibits inflammatory reactions, helps restore immune balance and prevent tissue damage, making it a vital component in resolving inflammation.^46^

Increasing IL‐10 levels may enhance therapy for inflammatory disease due to its anti‐inflammatory properties.^47^ In severe AP, patients exhibit an increase in IL‐10 plasma levels,^48^ alongside increased IL‐6 and IL‐8 levels. However, IL‐10 has a stronger correlation with outcomes than IL‐6 or IL‐8.^49^ This increase aligns with IL‐10's anti‐inflammatory role, which is essential for preventing tissue damage and modifying immune responses.^8^ Furthermore, increased anti‐inflammatory cytokines are part of the compensatory anti‐inflammatory response syndrome (CARS), marked by immune suppression and overproduction of cytokines.^50^ IL‐4 stimulates IL‐10 production, playing a crucial role in reducing tissue damage and promoting repair in experimental AP.^51^ Tirzepatide reduces inflammation by lowering pro‐inflammatory cytokines in diabetic neuropathy,^41^ and mitigates nephrotoxicity and neurotoxicity through a similar mechanism.^52^

Histopathological analysis revealed that L‐arginine‐treated rats exhibited gradual morphological changes in the pancreas, from tissue degradation to localized necrosis.^53^ Tirzepatide appears to mitigate these effects by enhancing insulin sensitivity and promoting cellular repair, potentially counteracting damage from conditions like pancreatitis.^54^ This aligns with earlier findings that activating GLP‐1 receptors, a key mechanism of Tirzepatide, promotes pancreatic health.^55^ By contrast, a prior study reported that the L‐arginine model for severe acute necrotizing pancreatitis shows a progression from necrosis to recovery, with acinar cell regeneration beginning after 7 days and nearly normal histology by day 14.^3^

It is noteworthy that L‐arginine activated the p‐Akt pathway, suggesting an adaptive response to stress.^56^ Tirzepatide regulates responses to stress and apoptosis by increasing Akt expression in L‐arginine‐induced pancreatitis. This finding of elevated p‐Akt in pancreatic tissues aligns with research on the PI3K/Akt pathway's involvement in promoting cell survival^57^ and may explain the elevated p‐Akt levels observed with Tirzepatide and L‐arginine co‐treatment compared to treatment with L‐arginine alone. Tirzepatide demonstrates notable anti‐inflammatory effects that contribute to improved cognitive function. Additionally, Tirzepatide effectively restored the PI3K/Akt/GSK3β pathway by normalizing aberrant changes in key molecules involved in inflammatory signaling pathways.^58^ Furthermore, Tirzepatide has shown neuroprotective effects by modulating the p‐Akt/CREB/BDNF pathway.^59^, ^60^

Tirzepatide demonstrates notable anti‐inflammatory effects that contribute to improved cognitive function. Additionally, Tirzepatide effectively restored the PI3K/Akt/GSK3β pathway by normalizing aberrant changes in key molecules involved in inflammatory signaling pathways.^58^ It is also well recognized that activation of GLP‐1 receptors stimulates the PI3K/AkT pathway, a key mediator of cytoprotective and anti‐inflammatory signaling. This mechanism is likely involved in the beneficial effects demonstrated in this study.^61^

The sustained activation of Akt following Tirzepatide treatment highlights its pivotal role in facilitating the transition from inflammation to tissue repair and regeneration while reducing pro‐inflammatory cytokines. Tirzepatide is able to enhance cell survival and promote tissue repair by maintaining Akt activity and inhibiting apoptosis. However, the potential long‐term effects, such as fibrosis and abnormal tissue growth, warrant further investigation. Future studies are crucial to comprehensively elucidate these pathways and evaluate the therapeutic potential of Tirzepatide in managing inflammatory pancreatic disorders, ultimately opening the door to more specific and effective interventions.

## 5 CONCLUSIONS

Tirzepatide significantly alleviates L‐arginine‐induced pancreatic damage by reducing inflammatory biomarkers, such as IL‐6, and oxidative stress, besides enhancing cytokines that suppress inflammation, such as IL‐4 and IL‐10. Furthermore, Tirzepatide promotes cellular repair by activating the p‐Akt signaling pathway. These combined anti‐inflammatory and antioxidant effects underscore its potential as a promising therapeutic agent for managing inflammatory disorders.

Tirzepatide demonstrates a combination of anti‐inflammatory and antioxidant properties, accompanied by a cellular repair mechanism, making it a promising therapeutic option in pancreatitis.

## AUTHOR CONTRIBUTIONS

**Razan Alawaji:** Conceptualization; data curation; formal analysis; funding acquisition; investigation; methodology; project administration; resources; software; validation; visualization; writing – original draft; writing – review and editing. **Mohamed S. Abdel‐Bakky:** Data curation; formal analysis; investigation; methodology; project administration; resources; writing – review and editing. **Hussein M. Ali:** Data curation; formal analysis; investigation; resources; software; validation. **Miad A. Aljuhani:** Investigation; methodology; resources. **Abdulaziz Arif A. Alshammari:** Data analysis; interpretation of results; visualization of findings; manuscript revision. **Hashim K. Kamal:** Investigation. **Maamoun M. K. Khoja:** Resources. **Kholoud Alsehemi:** Investigation. **Mennatallah A. Korani:** Data curation. **Eman S. Said:** Data curation; formal analysis; investigation; methodology; project administration; supervision; validation; visualization; writing – review and editing.

## FUNDING INFORMATION

No funding was received for this work.

## CONFLICT OF INTEREST STATEMENT

The authors declare no conflicts of interest.

## ETHICS STATEMENT

The animal study protocol was approved by the Ethics Committee of Qassim University (24‐01‐04) on August 19, 2024.

## INFORMED CONSENT STATEMENT

Not applicable.

## Data Availability

The datasets generated during and/or analyzed during the current study are not publicly available but are available from the corresponding author upon reasonable request.
